# Chronic Kidney Disease and Superimposed Acute Kidney Injury: Greater Impact of Acute Insults on Outcomes

**DOI:** 10.1155/ijne/1353892

**Published:** 2026-01-09

**Authors:** Mariana Wernersbach Chagas, Dayana Bitencourt Dias, Welder Zamoner, Daniela Ponce

**Affiliations:** ^1^ Botucatu School of Medicine, University of São Paulo State (UNESP), Ave. Prof. Mário Rubens Guimarães Montenegro, Botucatu, 18618687, São Paulo, Brazil

**Keywords:** acute kidney injury, acute kidney support, chronic kidney disease, mortality, risk factors

## Abstract

**Introduction:**

Acute kidney injury (AKI) and chronic kidney disease (CKD) are widely correlated. However, the risk factors associated with outcomes of AKI in CKD patients have not been widely studied to date.

**Objectives:**

To identify factors associated with outcomes of death and need for kidney support therapy (KST) in patients with CKD who present with AKI during hospital stay.

**Methods:**

Retrospective cohort conducted from July 2018 to June 2022 that included patients with CKD and superimposed AKI. Sociodemographic data related to CKD, AKI, and the progression of patients to outcomes as death and KST were collected. The results were discussed with a significance level of *p* < 0.05.

**Results:**

A total of 327 patients were included. The patients had a mean age of 68.6 ± 11.4 years, the majority were men, and the most prevalent comorbidities were hypertension (81.7%) and cardiovascular disease (61.5%). The mean creatinine was 1.85 ± 0.74 mg/dL. The main etiology of CKD was undetermined (26.6%) and of AKI was septic (45.3%). Patients were hospitalized mainly for infectious or cardiovascular causes (22.3% each). Overall mortality was 29.1%, and the need for KST was 35.2%. In the intensive care unit (ICU), 73.2% required dialysis and 74.4% died, reaching 85.7% in those with KST. CKD staging was not associated with any of the primary outcomes. The risk factors for KST were obesity, ATN‐ISS score, and creatinine elevation greater than three times the baseline. The risk factors for death were ATN‐ISS score, undetermined CKD, septic AKI, ICU admission, and KST.

**Conclusions:**

Mortality and need for KST in CKD patients admitted to the ICU and who develop AKI are high. Variables related to AKI were more relevant than those related to CKD for clinical outcomes.

## 1. Introduction

Acute kidney injury (AKI) is defined as an increase in serum creatinine greater than 0.3 mg/dL in 48 h, an increase in serum creatinine greater than 1.5 times baseline in 7 days, or a urine output < 0.5 mL/kg/h in 6 h [1]. The incidence of AKI varies depending on the definition used, patient characteristics, and the severity of the clinical scenario, but ranges from around 35%–40% to 78.7% [2]. Regarding outcomes, the need for kidney support therapy (KST) can reach 59.2% and mortality 80% [3].

Chronic kidney disease (CKD) is defined as a kidney abnormality (either functional or structural) present for more than 3 months [4]. Worldwide, it is estimated that around 650 to 900 million people have CKD, which was the cause of 1.43 million deaths in 2019 [5, 6]. In Brazil, the prevalence of CKD is uncertain, but there are data from the ELSA‐Brasil cohort indicating a prevalence of 8.9% [7].

CKD and AKI are widely correlated, with high prevalence and mortality and consequent impact on global health.

AKI is associated with a higher risk of progression to CKD. The meta‐analysis by See et al. showed that patients with AKI had a 2.67‐fold higher risk (95% CI 1.99–3.58, *p* < 0.001) of progressing to CKD [8]. A recent Danish retrospective cohort study identified that 26.3% to 29.5% of patients who had AKI (depending on AKI duration) progressed to CKD with up to 20 years follow‐up [9].

Similarly, patients with CKD have a higher risk of AKI episodes. The cohort of Hsu et al. identified that among patients who developed AKI, the majority had an estimated glomerular filtration rate (eGFR) < 60 mL/min/1.73 m^2^ (74%), with higher stages of CKD having a higher risk of AKI (stage 3a OR 1.95, 95% CI 1.66–2.3; stage 3b OR 6.54, 95% CI 5.57–7.69; stage 4 OR 28.5, 95% CI 24.5–33.14; stage 5 OR 40.07, 95% CI 33.75–47.58) [10].

In terms of outcomes, the literature data are controversial. Khosla et al., in the PICARD study, concluded that patients with CKD had greater dependence on dialysis (45% vs. 32%, *p* 0.06), but lower in‐hospital mortality (31% vs. 40%, *p* 0.04) [11]. The cohort of Lafrance et al. studied patients with CKD and identified worse outcomes in those with eGFR > 20 mL/min/1.73 m^2^ (RR death 2.52, 95% CI 2.17–2.94 and RR dialysis 4.88, 95% CI 4.08–5.84) compared with eGFR between 10 and 20 mL/min/1.73 m^2^ (RR death 2.0, 95% CI 1.56–2.55 and RR dialysis 1.28, 95% CI 1.07–1.53) [12].

There are still few studies that have evaluated the population with AKI and CKD, especially in developing countries, in addition to presenting controversial results. The objective of this study is to identify factors associated with outcomes of death and need for KST in patients with CKD who present with AKI during hospital stay.

## 2. Methods

A retrospective observational cohort study was carried out based on activation of the AKI Group to evaluate patients admitted to Botucatu School of Medicine University Hospital, São Paulo, Brazil, from January 2018 to June 2022. Data collection was performed by only one researcher, using the institutional and AKI Group electronic information systems. The study was conducted in observance of the Declaration of Helsinki and was approved by the local Research Ethics Committee of Botucatu School of Medicine University Hospital, under number 5,510,384 and Certificate of Presentation of Ethical Appreciation from Plataforma Brasil [Brazil Platform] (CAAE, acronym in Portuguese), under number 59641422.1.0000.5411.

To be eligible for the study, patients had to be over 18 years old, have CKD as a previous comorbidity, and present AKI during hospitalization. Pregnant patients, those undergoing palliative care, those without known baseline creatinine, those undergoing renal replacement therapy (transplant or dialysis) prior to hospitalization, or those lost to follow‐up (transfer or evasion) were excluded.

Patient identification data (age, sex, race, education), previous comorbidities, baseline creatinine, outpatient follow‐up with a nephrologist (including care indicators), cause of hospital admission, etiology and stage of CKD, etiology of AKI, need for intensive care unit (ICU), creatinine evolvement, as well as prognostic scores (APACHE II and ATN‐ISS) and outcomes (KST and death) were collected. Among the care indicators, serum laboratory data for hemoglobin, parathyroid hormone (PTH), bicarbonate and glycated hemoglobin (HbA1c) were collected, in addition to blood pressure (BP) data. Data related to laboratory tests were obtained from the most recent outpatient exam and BP measurement at the last appointment. For the KST outcome, data related to the indication and the therapy modality were collected. KST implementation was based on urgent dialysis (refractory hyperkalemia, refractory acidosis, overload, or uremia) and demand versus capacity gap. This last criterion is used when the metabolic and fluid demands on a patient’s kidneys exceed the kidneys’ functional capacity, before urgency criteria, and is an individualized decision making [13].

Both CKD and AKI were defined according to KDIGO criteria [1, 4]. CKD was considered in the presence of functional or structural abnormalities for at least 3 months. Baseline creatinine was considered the lowest outpatient creatinine in the last 6 months, with eGFR calculated using the CKD‐EPI 2021 equation [14]. AKI was evaluated at nephrologist activation. We defined AKI as one of the following: increase in serum creatinine by ≥ 0.3 mg/dL within 48 h or ≥ 1.5 times baseline within the prior 7 days. Obesity was defined as a body mass index (BMI) > 30 kg/m^2^. Good glycemic control was determined as HbA1c < 8% for all, although ideally the Brazilian Diabetes Society recommends different goals depending on the patient’s profile [15]. A good BP control is BP < 130/80 mmHg, according to the 2020 Brazilian Hypertension Guideline [16]. The APACHE II score was obtained within the first 24 h of ICU admission and the ATN‐ISS score on the first day of the nephrologist’s evaluation [17, 18].

Based on the study protocol, a descriptive analysis of the information obtained was performed. The need for KST and the occurrence of death were then established as dependent variables. We use IBM SPSS 29 and SigmaPlot 15 as statistical softwares for data analysis. Univariate statistical analysis was performed using the chi‐square test for categorical variables, the *t*‐test for continuous, parametric, and independent variables, and the Mann–Whitney test for continuous, nonparametric, and independent variables. Stepwise logistic regression was also performed, using only the statistically significant variables associated with death or the need for KST identified in the univariate analysis and that were noncollinear. The data were discussed with a 5% significance level and an 80% statistical power of the test.

## 3. Results

**Figure 1:**
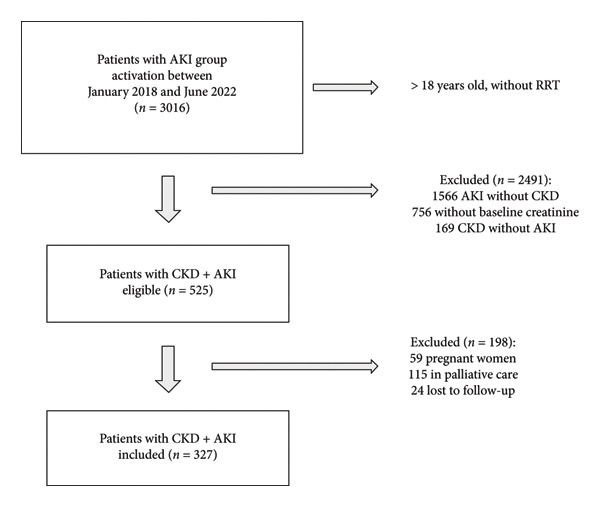
Flowchart of inclusion criteria. AKI, Acute kidney injury; CKD, Chronic kidney disease; RRT, Renal replacement therapy.

A total of 327 patients were included in the study. Figure 1 shows the flowchart of inclusion criteria. Initially, 3016 patients were identified, of which 525 were eligible. Of these, 198 were excluded.

It was identified that 60.9% were men, 89.6% White, with a mean age of 68.6 ± 11.4 years, and 67.1% had not completed elementary education. The most prevalent comorbidities were hypertension, cardiovascular disease, and diabetes (81.7%, 61.5%, and 51.7%, respectively), with 81% of patients having at least three comorbidities, as shown in Table 1.

**Table 1:** Demographic characteristics and comorbidities of patients with CKD and superimposed AKI regarding the outcome need for KST and death.

	Total (*n* = 327)	KST (*n* = 115)	Without KST (*n* = 212)	*p*	Deaths (*n* = 95)	Survivors (*n* = 232)	*p*
Age (years)	68.6 ± 11.4	69.1 ± 10.3	68.4 ± 12.0	0.584	70.4 ± 10.7	67.9 ± 11.6	0.070
Sex				0.234			0.963
Male	199 (60.9%)	75 (65.2%)	124 (58.5%)		58 (61.1%)	141 (60.8%)	
Female	128 (39.1%)	40 (34.8%)	88 (41.5%)		37 (38.9%)	91 (39.2%)	
Race				0.840			0.897
White	293 (89.6%)	104 (90.4%)	189 (89.2%)		86 (90.5%)	207 (89.2%)	
Mixed	22 (6.7%)	8 (7%)	14 (6.6%)		6 (6.3%)	16 (6.9%)	
Black	9 (2.8%)	2 (1.7%)	7 (3.3%)		2 (2.1%)	7 (3%)	
Asian	1 (0.3%)	0 (0%)	1 (0.5%)		0 (0%)	1 (0.4%)	
Education				0.376			0.304
Illiterate	10 (3.1%)	5 (4.4%)	5 (2.4%)		5 (5.3%)	5 (2.2%)	
Only literate	28 (8.6%)	11 (9.8%)	17 (8.2%)		10 (10.6%)	18 (8%)	
Incomplete elementary	181 (55.4%)	58 (51.8%)	123 (59.4%)		49 (52.1%)	132 (58.6%)	
Complete elementary	39 (11.9%)	12 (10.7%)	27 (13%)		9 (9.6%)	30 (13.4%)	
Complete high school	47 (14.4%)	22 (19.6%)	25 (12.1%)		18 (19.1%)	29 (12.8%)	
Complete higher education	14 (4.3%)	4 (3.6%)	10 (4.8%)		3 (3.2%)	11 (4.9%)	
Comorbidities							
Cardiovascular disease	201 (61.5%)	69 (60%)	132 (62.3%)	0.688	63 (66.3%)	138 (59.5%)	0.249
Arterial hypertension	267 (81.7%)	96 (83.5%)	171 (80.7%)	0.530	74 (77.9%)	193 (83.2%)	0.261
Diabetes	169 (51.7%)	59 (51.3%)	110 (51.9%)	0.920	44 (46.3%)	125 (53.9%)	0.214
Obesity	102 (31.2%)	44 (38.3%)	58 (27.4%)	0.042	32 (33.7%)	70 (30.2%)	0.534
Dyslipidemia	101 (30.9%)	43 (37.4%)	58 (27.4%)	0.080	30 (31.6%)	71 (30.6%)	0.862
Neoplasia	64 (19.6%)	18 (15.7%)	46 (21.7%)	0.188	15 (15.8%)	49 (21.1%)	0.270
Smoking history	151 (46.2%)	52 (45.2%)	99 (46.7%)	0.798	45 (47.4%)	106 (45.7%)	0.782
Urological	78 (23.9%)	26 (22.6%)	52 (24.5%)	0.697	18 (18.9%)	60 (25.9%)	0.183
Respiratory	30 (9.2%)	13 (11.3%)	17 (8%)	0.326	11 (11.6%)	19 (8.2%)	0.335
Hepatic	15 (4.6%)	8 (7%)	7 (3.3%)	0.131	8 (8.4%)	7 (3%)	0.034
Endocrinological	53 (16.2%)	17 (14.8%)	36 (17%)	0.606	18 (18.9%)	35 (15.1%)	0.390
Neurological	7 (2.1%)	3 (2.6%)	4 (1.9%)	0.667	3 (3.2%)	4 (1.7%)	0.416
Rheumatological	6 (1.8%)	1 (0.9%)	5 (2.4%)	0.338	2 (2.1%)	4 (1.7%)	0.816
Infectious	6 (1.8%)	2 (1.7%)	4 (1.9%)	0.924	3 (3.2%)	3 (1.3%)	0.254
Number of comorbidities				0.745			0.522
None	1 (0.3%)	0 (0%)	1 (0.5%)		0 (0%)	1 (0.4%)	
1 comorbidities	18 (5.5%)	5 (4.3%)	13 (6.1%)		6 (6.3%)	12 (5.2%)	
2 comorbidities	43 (13.1%)	14 (12.2%)	29 (13.7%)		16 (16.8%)	27 (11.6%)	
3 or more comorbidities	265 (81%)	96 (83.5%)	169 (79.7%)		73 (76.8%)	192 (82.8%)	

Abbreviations: CKD, chronic kidney disease; KST, kidney support therapy.

Regarding CKD, the mean creatinine was 1.85 ± 0.74 mg/dL and the eGFR was 41.5 ± 18.2 mL/min. The main etiologies were unknown, diabetic kidney disease, multifactorial, and hypertensive nephrosclerosis (26.6%, 18.7%, 16.2%, and 14.4%, respectively). Follow‐up with a nephrologist was performed by 42.2% of patients. Of these, the main etiologies of CKD were diabetes kidney disease and multifactorial (25.4% and 23.9%, respectively), 37.7% had albuminuria A3, 53.6% had BP < 130/80 mmHg, 79% HbA1c < 8% and, among the laboratory indicators, hemoglobin 11.8 ± 1.9 g/dL, PTH 148.8 ± 117.5 pg/dL, and bicarbonate 24.3 ± 3.6 mEq/L, as shown in Table 2.

**Table 2:** Characteristics of CKD in patients with CKD and superimposed AKI regarding the outcome need for KST and death.

	Total (*n* = 327)	KST (*n* = 115)	Without KST (*n* = 212)	*p*	Deaths (*n* = 95)	Survivors (*n* = 232)	*p*
Baseline creatinine (mg/dL)	1.85 ± 0.74	1.94 ± 0.88	1.80 ± 0.64	0.082	1.78 ± 0.7	1.88 ± 0.75	0.253
eGFR (mL/min)	41.5 ± 18.2	41.3 ± 19.8	41.6 ± 17.4	0.909	42.7 ± 17.4	41 ± 18.56	0.435
CKD stage				0.551			0.205
1	9 (2.8%)	5 (4.3%)	4 (1.9%)		3 (3.2%)	6 (2.6%)	0.774
2	24 (7.3%)	7 (6.1%)	17 (8%)		4 (4.2%)	20 (8.6%)	0.165
3a	96 (29.4%)	35 (30.4%)	61 (28.8%)		35 (36.8%)	61 (26.3%)	0.057
3b	108 (33%)	33 (28.7%)	75 (35.4%)		31 (32.6%)	77 (33.2%)	0.922
4	84 (25.7%)	32 (27.8%)	52 (24.5%)		22 (23.2%)	62 (26.7%)	0.503
5	6 (1.8%)	3 (2.6%)	3 (1.4%)		0 (0%)	6 (2.6%)	0.114
CKD etiology				0.453			< 0.001
Unknown	87 (26.6%)/11 (8%)^∗^	35 (30%)	52 (24.5%)		43 (45.3%)	44 (19%)	< 0.001
Hypertensive nephrosclerosis	47 (14.4%)/25 (18.1%)^∗^	18 (15.7%)	29 (13.7%)		14 (14.7%)	33 (14.2%)	0.904
Diabetic kidney disease	61 (18.7%)/35 (25.4%)^∗^	16 (13.9%)	45 (21.2%)		10 (10.5%)	51 (22%)	0.016
Glomerular	9 (2.8%)/8 (5.8%)^∗^	3 (2.6%)	6 (2.8%)		2 (2.1%)	7 (3%)	0.647
Obstructive	16 (4.9%)/2 (1.5%)^∗^	5 (4.3%)	11 (5.2%)		2 (2.1%)	14 (6%)	0.135
Ischemic	15 (4.6%)/6 (4.3%)^∗^	3 (2.6%)	12 (5.7%)		1 (1.1%)	14 (6%)	0.051
Tubulointerstitial	7 (2.1%)/4 (2.9%)^∗^	4 (3.5%)	3 (1.4%)		2 (2.1%)	5 (2.2%)	0.977
Sequelae of AKI	8 (2.4%)/5 (3.6%)^∗^	1 (0.9%)	7 (3.3%)		2 (2.1%)	6 (2.6%)	0.798
Multifactorial	53 (16.2%)/33 (23.9%)^∗^	20 (17.4%)	33 (15.6%)		13 (13.7%)	40 (17.2%)	0.428
Others	24 (7.3%)/9 (6.5%)^∗^	10 (8.7%)	14 (6.6%)		6 (6.3%)	18 (7.8%)	0.650
Follow up with nephrologist	138 (42.2%)	49 (42.6%)	89 (42%)	0.913	40 (42.1%)		0.982
Hemoglobin (gr/dL)^∗^	11.8 ± 1.9	12.1 ± 2.1	11.6 ± 1.8	0.212	12.4 ± 1.8	11.5 ± 1.9	0.022
PTH (pg/dL)^∗^	148.8 ± 117.5	136.4 ± 77.9	155.1 ± 133.4	0.403	131.6 ± 84.6	155.5 ± 128	0.310
Bicarbonate (mEq/L)^∗^	24.3 ± 3.6	24.3 ± 4.4	24.3 ± 3.1	0.988	24.6 ± 4.3	24.1 ± 3.3	0.567
BP < 130/80 mmHg^∗^	74 (54%)	31 (63.3%)	43 (48.9%)	0.105	24 (60%)	50 (51.5%)	0.367
HbA1c < 8%^∗^	50 (64.1%)	18 (64.3%)	32 (64%)	0.980	13 (59.1%)	37 (66.1%)	0.563
Albuminuria^∗^				0.075			0.110
1	39 (29.3%)	12 (25%)	27 (31.8%)		13 (33.3%)	26 (27.7%)	
2	42 (31.6%)	21 (43.8%)	21 (24.7%)		16 (41%)	26 (27.7%)	
3	52 (39.1%)	15 (31.2%)	37 (43.5%)		10 (25.6%)	42 (44.7%)	

Abbreviations: BP, Blood pressure; CKD, Chronic kidney disease; HbA1c, Glycated hemoglobin; KST, Kidney support therapy; PTH, Parathyroid hormone.

^∗^Followed by nephrologist.

In relation to AKI, the most common etiologies were septic and ischemic (45.3% and 31.5%, respectively). At the time of activation of the AKI Group, the mean creatinine was 3.7 ± 2.3 mg/dL, while the maximum value reached 4.7 ± 2.6 mg/dL. The main causes of hospitalization were infection and cardiovascular disease (22.3% both). The length of hospitalization was 14.9 ± 12.6 days, and 26.3% of patients were admitted to the ICU. Among the prognostic scores, the ATN‐ISS was 0.32 ± 0.2 and the APACHE II for ICU patients was 21.5 ± 6.7, as shown in Table 3.

**Table 3:** Characteristics of hospitalization and AKI in patients with CKD and superimposed AKI regarding the outcome need for KST and death.

	Total (*n* = 327)	KST (*n* = 115)	Without KST (*n* = 212)	*p*	Deaths (*n* = 95)	Survivors (*n* = 232)	*p*
AKI etiology							
Ischemic	103 (31.5%)	23 (20%)	79 (37.3%)	0.001	15 (15.8%)	87 (37.5%)	< 0.001
Septic	148 (45.3%)	58 (50.4%)	76 (35.8%)	0.015	60 (63.2%)	74 (31.9%)	< 0.001
COVID‐19 associated	14 (4.3%)	12 (10.4%)	2 (0.9%)	< 0.001	12 (12.6%)	2 (0.9%)	< 0.001
Obstructive	15 (4.6%)	2 (1.7%)	13 (6.1%)	0.070	0 (0%)	15 (6.5%)	0.011
Nephrotoxic	16 (4.9%)	5 (4.3%)	11 (5.2%)	0.736	0 (0%)	16 (6.9%)	0.009
Glomerular	1 (0.3%)	0 (0%)	1 (0.5%)	0.461	0 (0%)	1 (0.4%)	0.522
Multifactorial	44 (13.5%)	14 (12.2%)	30 (14.2%)	0.617	7 (7.4%)	37 (15.9%)	0.039
Creatinine rise				< 0.001			0.022
Up to 2 times basal creatinine	141 (43.1%)	31 (26.9%)	110 (51.8%)	< 0.001	30 (31.6%)	111 (78.9%)	0.007
Between 2 and 3 times basal creatinine	88 (26.9%)	28 (24.3%)	60 (28.3%)	0.441	29 (30.5%)	59 (25.4%)	0.346
Greater than 3 times basal creatinine	98 (30%)	56 (48.7%)	42 (19.8%)	< 0.001	36 (37.9%)	62 (26.7%)	0.045
Creatinine on activation (mg/dL)	3.7 ± 2.3	4.49 ± 2.8	3.39 ± 1.92	< 0.001	3.49 ± 1.85	3.9 ± 2.49	0.148
Maximum creatinine (mg/dL)	4.7 ± 2.6	5.9 ± 2.78	4.1 ± 2.33	< 0.001	4.69 ± 2	4.75 ± 2.86	0.863
Need for KST	115 (35.2%)	—	—	—	70 (73.7%)	45 (19.4%)	< 0.001
KST indication							0.985
Demand versus capacity gap	87 (75.7%)	—	—	—	53 (75.7%)	34 (75.6%)	
Urgent	28 (24.3%)	—	—	—	17 (24.3%)	11 (24.4%)	
KST modality							< 0.001
Conventional HD	57 (49.6%)	—	—	—	23 (32.9%)	34 (75.6%)	< 0.001
Prolonged HD	35 (30.4%)	—	—	—	33 (47.1%)	2 (4.4%)	< 0.001
CVVT	1 (0.9%)	—	__	—	1 (1.4%)	0 (0%)	0.421
PD	15 (13%)	—		—	6 (8.6%)	9 (20%)	0.076
Without hemodynamic conditions	7 (6.1%)	—	—	—	7 (10%)	0 (0%)	0.029
Cause of hospitalization							
Elective surgery	46 (14.1%)	17 (14.8%)	29 (13.7%)	0.784	12 (12.6%)	34 (14.7%)	0.633
Cardiovascular	73 (22.3%)	23 (20%)	50 (23.6%)	0.457	18 (18.9%)	55 (23.7%)	0.348
COVID‐19	14 (4.3%)	12 (10.4%)	2 (0.9%)	< 0.001	12 (12.6%)	2 (0.9%)	< 0.001
Infection	73 (22.3%)	31 (27%)	42 (19.8%)	0.179	27 (28.4%)	46 (19.8%)	0.122
Urological	24 (7.3%)	5 (4.3%)	19 (9%)	0.127	1 (1.1%)	23 (9.9%)	0.005
Neurological	20 (6.1%)	8 (7%)	12 (5.7%)	0.640	10 (10.5%)	10 (4.3%)	0.033
Acute abdomen	43 (13.1%)	10 (8.7%)	33 (15.6%)	0.079	10 (10.5%)	33 (14.2%)	0.369
Others	34 (10.4%)	8 (7%)	19 (9%)	0.529	3 (3.2%)	24 (10.3%)	0.032
Time until nephrologist activation (days)	2 (1–5)	2 (1–5)	2 (1–6)	0.881	3 (1–5)	2 (1–5)	0.242
Time from nephrologist activation to KST (days)	1 (0–4)	—	—	—	1 (0–2)	3 (1–7.5)	0.001
ATN‐ISS score	0.32 ± 0.2	0.47 ± 0.24	0.25 ± 0.1	< 0.001	0.53 ± 0.23	0.24 ± 0.1	< 0.001
APACHE II score	21.5 ± 6.7	22.5 ± 6.7	18.8 ± 6	0.038	22.8 ± 6.5	17.3 ± 5.8	0.002
Length of hospitalization (days)	14.9 ± 12.6	14 (7–21)	11 (8–17)	0.101	12.4 ± 9.6	15.9 ± 13.5	0.02
ICU	86 (26.3%)	63 (54.8%)	23 (10.8%)	< 0.001	64 (67.4%)	22 (9.5%)	< 0.001
Length in the ICU (days)	5 (2–14)	8 (2–15)	5 (2–11)	0.327	5.5 (2–14)	5 (2.75–14)	0.551

Abbreviations: APACHE, Acute physiology and chronic health evaluation; ATN‐ISS, Acute tubular necrosis individual severity score; CKD, Chronic kidney disease; CVVT, Continuous venovenous therapy; HD, Hemodialysis; ICU, Intensive care unit; KST, Kidney support therapy; PD, Peritoneal dialysis.

The outcomes analyzed were death and need for KST. Overall mortality was 29.1%, and the need for dialysis was 35.2%. Among patients in KST, the main indication was demand versus capacity gap (75.7%) and the most prescribed modality was conventional hemodialysis (HD) (49.6%). Of the patients in the ICU, 73.2% required KST and 74.4% died. Mortality among patients in KST was 60.8%, reaching 85.7% in the ICU, as shown in Table 3.

Table 1 shows the demographic characteristics and comorbidities regarding the outcomes of death and KST.

Concerning the need for KST, obesity was the only variable associated with the risk of KST (38.3 vs. 27.4%, *p* = 0.042).

Regarding death, hepatic comorbidity was the only variable associated with the risk of death (8.4 vs. 3%, *p* = 0.034).

Table 2 shows the characteristics related to CKD in relation to the outcomes studied.

In relation to the KST outcome, the mean creatinine, stage, etiology, follow‐up with a nephrologist, care indicators, and eGFR were similar between the groups.

Regarding death, unknown cause of CKD was more frequent in patients who died (45.3 vs. 19%, *p* < 0.001), and this population also had higher hemoglobin levels at hospital admission (12.4 ± 1.8 g/dL vs. 11.5 ± 1.9 g/dL, *p* = 0.022). Diabetic kidney disease was less prevalent in patients who died (22 vs. 10.5%, *p* = 0.016).

Table 3 shows the characteristics of hospitalization and AKI regarding unfavorable outcomes.

Regarding the need for KST, the KST group had a higher prevalence of patients with septic AKI (50.4 vs. 35.8%, *p* = 0.015) and associated with COVID‐19 (10.4 vs. 0.9%, *p* < 0.001) and a lower prevalence of ischemic AKI (20 vs. 37.3%, *p* = 0.001) compared to the group without KST. Hospitalization due to COVID‐19 was more frequent in the KST group (10.4 vs. 0.9%, *p* < 0.001). During hospitalization, while an increase of at least 3 times in baseline serum creatinine was more prevalent in the population that required dialysis (48.7 vs. 19.8%, *p* < 0.001), an increase of up to 2 times was more common in the group without KST (26.9 vs. 51.8%, *p* = 0.008). Both the mean creatinine at nephrology activation and the maximum creatinine were higher in the dialysis group (4.49 ± 2.8 mg/dL vs. 3.39 ± 1.92 mg/dL, *p* < 0.001 and 5.9 ± 2.78 mg/dL vs. 4.1 ± 2.33 mg/dL, *p* < 0.001, respectively). Both APACHE II and ATN‐ISS scores were higher in the KST group compared to the non‐KST group (22.5 ± 6.7 vs. 18.8 ± 6, *p* = 0.038 and 0.47 ± 2.8 vs. 0.25 ± 0.1, *p* < 0.001, respectively). The need for ICU also prevailed in patients who required dialysis (54.8 vs. 10.8%, *p* < 0.001).

Concerning the death outcome, the ischemic, obstructive, nephrotoxic, and multifactorial etiologies of AKI were more prevalent in surviving patients (15.8 vs. 37.5%, *p* < 0.001; 0 vs. 6.5%, *p* = 0.011; 0 vs. 6.9%, *p* = 0.009; and 7.4 vs. 15.9%, *p* = 0.039, respectively), while septic and associated with COVID‐19 were more frequent in the death group (63.2 vs. 31.9%, *p* < 0.001; 12.6 vs. 0.9%, *p* < 0.001). Hospitalization due to COVID‐19 occurred more frequently in the death group (12.6 vs. 0.9%, *p* < 0.001), while urological, neurological, and other causes were more prevalent in the survivor group (1.1 vs. 9.9%, *p* = 0.005; 10.5 vs. 4.3%, *p* = 0.033; and 3.2 vs. 10.3%, *p* = 0.032, respectively). Both prognostic scores, APACHE II and ATN‐ISS, were higher in the group that died (22.8 ± 6.5 vs. 17.3 ± 5.8, *p* = 0.002 and 0.53 ± 0.23 vs. 0.24 ± 0.1, *p* < 0.001, respectively). Other factors that were more prevalent in the death group were admission to the ICU (67.4 vs. 9.5%, *p* < 0.001) and shorter time between specialist call and indication of KST (1 (0–2) vs. 3 (1–7.5) days, *p* = 0.001). On the other hand, patients who died had a shorter hospital stay compared to those who were discharged (12.4 ± 9.6 days vs. 15.9 ± 13.5 days, *p* = 0.02). Dialysis therapy was more frequent in the death group (73.7 vs. 19.4%, *p* < 0.001) and, when indicated, not being able to start therapy and prolonged HD modality were more prevalent in patients who died (10 vs. 0%, *p* = 0.029 and 47.1 vs. 4.4%, *p* < 0.001, respectively), while conventional HD modality was more common in the group that was discharged from hospital (32.9 vs. 75.6%, *p* < 0.001). Regarding the severity of AKI, patients who had an increase of at least 3 times the baseline creatinine were more prevalent in the group that evolved to death (37.9 vs. 26.7%, *p* = 0.045), while the group that had an increase of up to 2 times the baseline creatinine was more frequent in the surviving population (31.6 vs. 78.9%, *p* = 0.007).

When backward stepwise logistic regression was performed, only the variables obesity (OR 5.1, 95% CI 1–25.9, *p* = 0.05), ATN‐ISS score (OR 146.8, 95% CI 7.2–2971.8, *p* = 0.001) and creatinine rise greater than three times the baseline value (OR 4.5, 95% CI 1.02–19.7, *p* = 0.046) were risk factors for the need for KST. ICU admission (OR 6.5, 95% CI 2.9–14.5, *p* < 0.001), ATN‐ISS score (OR 214.9, 95% CI 25.75–1794.6, *p* < 0.001), need for KST (OR 3.5, 95% CI 1.6–7.8, *p* = 0.002), CKD of unknown etiology (OR 2.3, 95% CI 1.04–5.21, *p* = 0.04) and AKI of septic etiology (OR 2.8, 95% CI 1.33–6, *p* = 0.007) were identified as risk factors for mortality, as shown in Table 4.

**Table 4:** Multivariate logistic regression for predictors of unfavorable outcomes.

	OR	95% CI	*p*
KST predictors			
Obesity	5.1	1.002–25.9	0.050
ATN‐ISS score	146.8	7.2–2971.8	0.001
APACHE II score	1.1	0.1–1.3	0.055
Ischemic AKI	0.3	0.05–1.4	0.114
Creatinine rise greater than three times the baseline value	4.5	1.02–19.7	0.046
Death predictors			
ATN‐ISS score	214.9	25.75–1794.6	< 0.001
Hepatic comorbidity	3.9	0.96–15.8	0.056
Unknown CKD	2.3	1.04–5.21	0.04
ICU	6.5	2.9–14.5	< 0.001
Septic AKI	2.8	1.33–6	0.007
KST	3.5	1.6–7.8	0.002

Abbreviations: AKI, Acute kidney injury; APACHE, Acute physiology and chronic health evaluation; ATN‐ISS, Acute tubular necrosis individual severity score; CI, Confidence interval; CKD, Chronic kidney disease; ICU, Intensive care unit; KST, Kidney support therapy; OR, Odds ratio.

## 4. Discussion

More than 3000 patients followed by the Botucatu School of Medicine University Hospital IRA Group were evaluated, but more than 2000 were excluded because they did not have CKD or known baseline creatinine.

Khosla et al., when studying patients with CKD and superimposed AKI, identified mortality similar to that found in the present study (31% and 29.1%, respectively), but differed in the prevalence of KST (61% and 35.2%, respectively) [11]. Acosta‐Ochoa et al., in turn, identified similar baseline creatinine (1.9 vs. 1.85 mg/dL), but lower need for dialysis and mortality (14 vs. 35.2% and 23 vs. 29.1%) [19].

We intuitively think that more advanced CKD stages would have worse outcomes when superimposed on AKI, but the literature is controversial. Khosla et al. concluded that CKD patients had greater dependence on dialysis but lower mortality [11]. Lafrance et al. identified worse outcomes in CKD patients with less advanced stages [12]. Therefore, a possible reason for this conclusion could be the adapted mechanisms of the hypoxic microenvironment as ischemic preconditioning [20].

This study identified obesity, ATN‐ISS score, and a 3‐fold increase in baseline creatinine as factors associated with the need for KST. Factors associated with death were CKD of unknown etiology, need for ICU and KST, ATN‐ISS score, and septic AKI.

The literature suggests a reverse epidemiology of obesity in CKD, in which there would be greater survival in patients with higher BMI, especially in advanced stages and in RRT [21]. On the other hand, this comorbidity appears to be associated with a higher risk of AKI and KST, according to a retrospective cohort of ICU patients with sepsis [22]. Although no association was identified between obesity and mortality, the present study corroborates the literature by showing that obesity was associated with the need for KST.

Although the literature has validated the ATN‐ISS score as a predictor of mortality since the 1990s, the present study identified this score as a predictor of both death and KST, as Liaño et al. demonstrated in the population with nonoliguric acute tubular necrosis [18, 23]. The ATN‐ISS score uses 9 variables, of which advanced age, male sex, oliguria, hypotension, jaundice, coma, and assisted respiration increase the score value, conferring a worse prognosis, while having nephrotoxicity as the main etiology of AKI reduces the score. In the study by Liaño et al., there was no survivor with a score greater than 0.9 (cutoff value with 100% specificity and 100% positive predictive value) [18]. Later, Balbi et al. validated this score in the Botucatu School of Medicine University Hospital, demonstrating mortality similar to that in the literature and reliability in clinical practice, providing mortality prediction, severity stratification, and an indicator of internal care [24].

Regarding the rise in creatinine, Pan et al. also observed that stage 3 AKI in patients with CKD was associated with a greater need for dialysis (87%, *p* < 0.001) [25].

CKD of unknown etiology has a variable prevalence, and in patients on chronic dialysis, it is present in 10% of cases [26]. In the present study, more than 25% of patients had unknown etiology of CKD. However, among the patients previously followed by nephrologists, more than 90% had a defined etiology. There are no robust studies on the prognosis of these patients, but the association found between unknown etiology and mortality is probably due to the impairment of one of the pillars of CKD treatment, which is the specific management of the underlying cause.

The need for ICU care as a predictor of mortality was also identified by Ponce et al. with a relative risk of 2.12 (95% CI 1.73–3.66, *p* = 0.003), even though it was in the general population with AKI [27]. The need for KST was also shown to be a predictor of mortality in the study by Pan et al., in which 56% of patients who died required KST compared to 26% of those who survived (*p* < 0.001) [25].

Concerning the etiology of AKI, in the Brazilian cohort of Pinheiro et al., 38% of ICU patients with septic AKI died compared to 16% in those with nonseptic AKI (*p* = 0.0087). Among patients with CKD and superimposed AKI, mortality was higher in those with septic AKI (39 vs. 0%, *p* = 0.001) [28]. Pan et al. also corroborated this association between septic AKI in ICU patients and mortality (48 vs. 75%, *p* = 0.019) [25]. In the present study, having septic AKI increased the risk of death by more than 2‐fold (OR 1.33–6, *p* = 0.007).

Although CKD and AKI are correlated, in the present study, clinical and renal insults that led to hospitalization and occurred during the in‐hospital period were more relevant than those related to CKD for the outcomes of need for KST and death. When it comes to the underlying disease, the role of CKD stages in unfavorable outcomes was already divergent in the literature [11, 12]. In this study, CKD staging was not associated with the primary outcomes, suggesting that care for hospitalized CKD patients should be similar, regardless of the stage they are in.

The prevalence of CKD in Brazil remains underestimated due to the lack of diagnosis. In this study, 25% of patients evaluated by the AKI Group in the proposed period did not have defined baseline creatinine, although more than 75% of patients had more than three comorbidities known to be risk factors for CKD. It is also necessary to discuss whether or not it is appropriate to extrapolate the AKI criteria to AKI superimposed on CKD.

This study has some limitations. Retrospective analysis and data collection from medical records depend on adequate documentation by the professionals responsible for direct care. Urine output was not used in the AKI assessment due to limited data source, which may have underestimated the incidence of AKI. CKD patients who presented with AKI without the need for nephrologist consultation were not included, which may have underestimated mild AKI. The study did not analyze the post–hospital discharge period and did not compare this population with patients with AKI without CKD. Nevertheless, these limitations are not sufficient to invalidate the results obtained.

Further studies are needed to compare the outcomes of the population with AKI and CKD with those of the population with AKI without CKD.

## 5. Conclusion

Among the studied population with CKD and superimposed AKI, there was a predominance of males, mean age of 68.4 years, with a high prevalence of arterial hypertension, predominance of CKD stage 3b of unknown etiology followed by diabetic kidney disease, AKI of septic etiology, with the need for ICU in 26.3% and for KST in 35.2% and overall mortality of 29.1%.

Risk factors for KST were obesity, ATN‐ISS score, and increase in baseline creatinine greater than three times the baseline value, while factors associated with death were CKD of unknown etiology, AKI of septic etiology, ICU admission, ATN‐ISS score, and the need for KST.

In this study, CKD staging was not associated with the outcomes of KST and death.

## Ethics Statement

The study was conducted in observance of the Declaration of Helsinki and was approved by the local Research Ethics Committee of Botucatu School of Medicine University Hospital, under number 5,510,384, and Certificate of Presentation of Ethical Appreciation from Plataforma Brasil [Brazil Platform] (CAAE, acronym in Portuguese), under number 59641422.1.0000.5411.

## Conflicts of Interest

The authors declare no conflicts of interest.

## Author Contributions

Mariana Wernersbach Chagas: conceptualization (lead), formal analysis (equal), methodology (equal), writing–original draft preparation (equal), and writing–review and editing (equal). Dayana Bitencourt Dias: methodology (supporting), writing–original draft preparation (supporting), and writing–review and editing (equal). Welder Zamoner: methodology (supporting), writing–original draft preparation (supporting), and writing–review and editing (equal). Daniela Ponce: conceptualization (supporting), formal analysis (equal), methodology (equal), writing–original draft preparation (equal), and writing–review and editing (equal).

## Funding

There was no funding received for this study.

## Data Availability

The data that support the findings of this study are not openly available due to reasons of sensitivity and are available from the corresponding author upon reasonable request.
